# Haplodiploidy accelerates mitogenome evolution in insects

**DOI:** 10.1098/rspb.2025.1813

**Published:** 2025-11-26

**Authors:** Avas Pakrashi, Ken A. Thompson, Paul D. N. Hebert

**Affiliations:** ^1^ University of Guelph, Centre for Biodiversity Genomics, Guelph, Ontario, Canada N1G 2W1

**Keywords:** rate acceleration, ploidy, DNA barcoding, molecular evolution, mito-nuclear interactions, COI

## Abstract

Rates of mitogenome evolution differ among animal lineages, and this variation has been linked to life history, to ecological traits and—potentially—to the sex-determination system. Insects are a compelling model for examining the latter factor because haplodiploid (HD) has evolved on multiple occasions from a diplodiploid (DD) ancestral state. We tested for rate differences between DD and HD taxa by examining sequence change in a sentinel segment of the mitogenome, the 658 bp barcode region of the cytochrome *c* oxidase subunit I (COI) gene. Specifically, we investigated if amino acid substitutions and indels are more frequent in HD than DD lineages by inspecting COI sequences from over 86 000 BINs (a species proxy) representing 783 insect families and 26 orders. Among them, 10 lineages, varying in rank from tribe to order, are HD. Our analysis, which accounts for phylogeny, indicates that HD lineages have higher rates (1.7×) of amino acid substitution, higher *K*_a_/*K*_s_ (3.5×) and far more indels than DD taxa. While our results demonstrate that HD accelerates mitogenome evolution, future work is needed to clarify its mechanistic basis. We hypothesize that HD facilitates positive selection for mitochondrial mutations which encode proteins that interact with nuclear gene products. Such coevolutionary interactions should be facilitated because recessive mutations in the nuclear genome are fully exposed to selection in males of the HD but not the DD lineages.

## 1. Introduction

Rates of mitogenome evolution differ markedly among animal lineages [1,2]. Prior investigations have linked this variation to flight loss, metabolic rate, body mass, generation length, and the evolution of a parasitic lifestyle [3–8]. However, one of the most striking observations of rate variation lacks explanation. Early phylogenetic analyses revealed accelerated mitogenome evolution in the honeybee [9]. While initially attributed to eusociality, similar acceleration was subsequently reported in solitary hymenopterans [10]. The hypothesis that their sex-determination system, haplodiploid (HD) versus diplodiploid (DD), might underpin rate acceleration was rejected owing to the difficulty in understanding how male haploidy could influence the evolution of the maternally inherited mitogenome (but see [11]). However, the potential for a linkage was reinvigorated when Li *et al*. [12] reported accelerated evolution of both mitochondrial and nuclear genes related to mitochondrial functions in five HD lineages of arthropods. The present study greatly extends the taxonomic scope of investigation by examining sequence variation in a mitochondrial gene among 783 families and nearly all HD lineages in the class Insecta.

**Figure 1.**
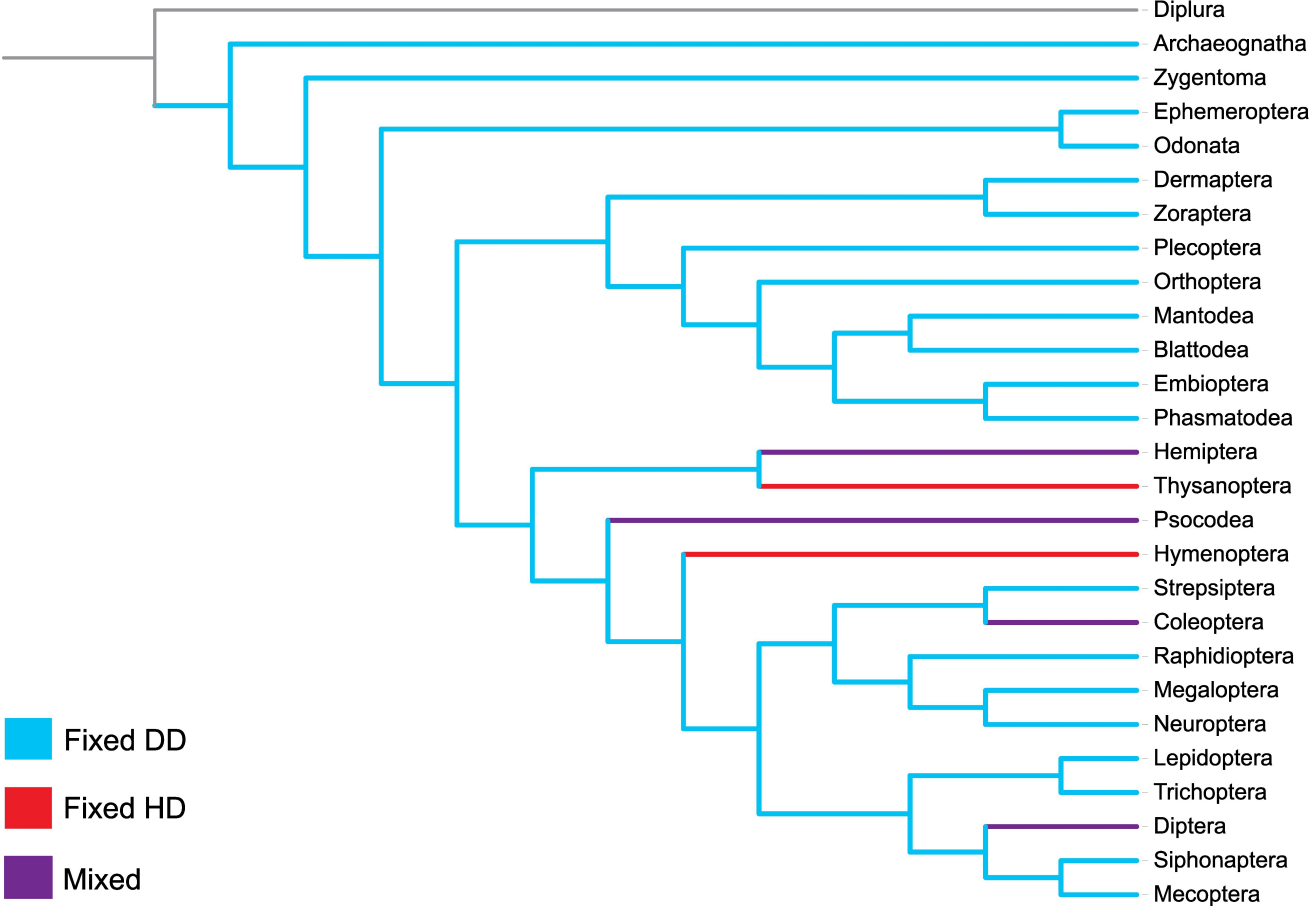
Macroevolutionary distribution of diplodiploidy (DD—blue) and haplodiploidy (HD—red) among insect orders. Orders containing both sex-determining systems are shown in purple, while the outgroup is in black.

Because HD, which encompasses both arrhenotoky (males develop from unfertilized eggs) and paternal genome elimination (paternal chromosomes are lost or inactivated during development), has evolved on multiple occasions in insects, the generality of rate acceleration in HD taxa can be tested in a comparative framework. All species in two orders (Hymenoptera and Thysanoptera) are HD, as are some lineages of Hemiptera, Psocodea, Diptera and Coleoptera [13–15] (figure 1). Within the Hemiptera, HD occurs in the Aleyrodidae (whiteflies) and in all 17 families of the Coccoidea (scale insects) [14,16,17]. In the Psocodea (bark, book and parasitic lice), HD occurs in the Liposcelididae and in several parasitic lice families (Phthiraptera) [18]. Within the Diptera, HD characterizes all Cecidomyiidae and some Sciaridae [19]. Finally, in the Coleoptera, HD occurs in the sole genus of Micromalthidae and in some species in two of the 19 tribes (Cryphalini, Xyloborini) of Scolytinae, one of the 19 subfamilies of Curculionidae [14,20,21].

Rigorous tests are available to examine rate variation; most consider the ratio of non-synonymous to synonymous substitutions (*K*_a_/*K*_s_) in a phylogenetic context (e.g. [22,23]). Rate acceleration can arise through positive or relaxed selection, and their discrimination is difficult because both generate similar sequence signatures. Given this fact, identification of the key factor underpinning acceleration in each case is typically inferential, based on consideration of differential exposure to genetic drift or selection. For example, rate acceleration in parasitic lineages has generally been attributed to positive selection [8], while its occurrence in flightless taxa has been attributed to relaxed selection linked to their low effective population sizes (*N*_e_) [7]. In other cases, such as the accelerated mitogenome evolution in high-elevation species, both explanations have been invoked [24]. HD and DD lineages are likely to be differentially exposed to drift and selection. For example, nuclear loci should be more exposed to drift in HD lineages because of their lower *N*_e_ [25,26]; but this difference does not extend to the mitogenome because of its maternal inheritance. Viewed from the perspective of positive selection [27], HD should facilitate the fixation of beneficial mutations involving nuclear–mitochondrial interactions because recessive mutations in the nuclear genome are fully exposed to selection in haploid males [11,28–30].

In this study, we consider patterns of sequence diversity in the barcode region of the mitochondrial cytochrome *c* oxidase subunit I (COI) gene [31] across the Insecta. Our analysis expands consideration from the five arthropod lineages examined by Li *et al*. [12] to 783 insect families in 26 orders. Although it spans just 5% of the mitogenome, the barcode region is a sentinel for mitogenome-wide shifts in nucleotide composition and evolutionary rate [32–34]. We examine three aspects of sequence diversity in COI: the incidence of amino acid substitutions, variation in *K*_a_/*K*_s_ and the frequency of indels. Aside from comparisons between the two purely HD orders (Hymenoptera, Thysanoptera) and the 20 purely DD orders, we compare HD and DD lineages in the four orders (Coleoptera, Diptera, Hemiptera and Psocodea) with mixed sex-determination systems. We acknowledge that family-level designations are not strictly uniform across insect orders; however, our conclusions do not depend on the objectivity of these ranks, but rather on relative comparisons between HD lineages and their DD counterparts.

## 2. Material and methods

### (a) Dataset preparation

We constructed a dataset from COI sequences that are publicly available on BOLD [35,36]. We downsampled from 16 million to 1.3 million records by randomly selecting a single sequence per BIN [37], only retaining sequences with complete coverage for the barcode amplicon (643–661 bp). This range, which results from deletions and insertions, spans all length variations so far detected in the barcode region of insects. We further reduced the dataset by employing stratified selection to help balance taxonomic representation. For families with <100 BINs, every BIN was included. For families with 100–1000 BINs, a random subset of 100 BINs was selected. In families with more than 1000 BINs, 100 were randomly chosen from each large subfamily (>100 BINs) together with all BINs for those subfamilies with <100. This approach produced a BOLD dataset (DS-INSECT1K) with a single representative of 87 434 BINs belonging to 928 families and 26 orders. Families with fewer than three BINs were subsequently excluded to prevent bias in downstream analyses—none of these families was HD or sister to HD clades. A separate BOLD dataset (DS-CURTRIBE) comprising 72 BINs was created for the family Curculionidae to ensure the widest possible representation of its subfamilies and tribes. This was required to evaluate the impact of HD in this family because only 2 of its 253 tribes [38] are known to employ this sex-determination system.

### (b) Sequence alignment and quality control

All sequences were aligned using MUSCLE v. 3.8.31 [39] with default parameters, implemented in AliView [40], to ensure consistency in codon positioning. The reading frame for all sequences was then manually verified to detect potential frameshift mutations or stop codons arising from sequencing errors or nuclear–mitochondrial DNA segments (NUMTs). Sequences containing misaligned codons were excluded to ensure high-quality data for downstream analyses. The final dataset comprised 86 548 BINs representing 783 families from 26 orders (electronic supplementary material, table S1). The nucleotide and amino acid alignments are available via Dryad (doi:10.5061/dryad.w0vt4b959) [41].

### (c) Substitution analysis of consensus amino acid sequences

A consensus nucleotide sequence was constructed for each family using the ‘most common base’ criterion in Geneious Prime 2025.1, and it was then translated into amino acids using the invertebrate mitochondrial code in MEGA11 [42]. Following the same approach, consensus amino acid sequences were generated for two orders of Entognatha (Diplura and Protura), the subphylum most closely related to the Insecta, to serve as reference ancestral sequences and as an outgroup for downstream phylogenetic analysis. The number of amino acid differences between the consensus sequence for each insect family and the outgroup sequences was computed in Geneious Prime. Data visualization was performed using radar plots and boxplots generated with plotly [43] and ggplot2 [44] in RStudio [45] using R v. 4.4.3 [46]. The pairwise amino acid distance between the consensus sequences for the families in each order was calculated using the *p*-distance metric in MEGA11. Statistical tests employed the XLMiner Analysis ToolPak in Microsoft Excel [47].

### (d) Variation in the ratio of non-synonymous to synonymous substitutions

We determined the median *K*_a_/*K*_s_ for each of the 783 families by calculating values for every pairwise comparison of their component BINs. We then employed ANOVA with type-II SS (note: the effect of HD versus DD was consistent when tested with type-III SS) to investigate the role of three independent variables (HD or DD; flight or flightless females; parasite or free-living) in explaining variation in *K*_a_/*K*_s_ among families using the R package ‘car’ [48]. Each family was assigned to a category using a majority rule basis. For example, it was only placed in the parasite/parasitoid category if >50% of its component species are believed to have this lifestyle [7,49–51]. Electronic supplementary material, table S2 shows the assignments for each family.

### (e) Variation in rates of amino acid substitution

To compare rates of molecular evolution in HD and DD taxa, we performed a phylogenetic analysis, which accounted for the lack of statistical independence of close relatives [52]. Its implementation required a phylogeny for all insect families. As none exists, we constructed one by compiling relevant data (electronic supplementary material, figure S1). Because statistical independence is determined by the topology of the tree, we ensured it was accurate at all levels where topology could affect our conclusions. The order-level topology was based on Misof *et al*. [53]. For orders with HD lineages, phylogenetic relationships were constrained to the best available topology as priors and constructed using BEAST 2.6.3 [54]. Within Hemiptera, HD lineages in the suborder Sternorrhyncha (Coccoidea and Aleyrodoidea) were constrained following the topology proposed by Song *et al*. [55]. A similar approach was applied to Psocodea [56] and Diptera [57]. For all other orders, neighbour-joining phylogenies were constructed and integrated within the respective orders using R Studio with the APE [58] and phytools [59] packages.

For phylogenetic analysis, we selected and aligned a single random sequence from each family. We tested for rate variation using RELAX v. 4.5 [23] implemented in HyPhy v. 2.5.64 [60]. RELAX provides a test for intensified or relaxed selection in a codon-based phylogenetic framework. We defined all HD clades as one subset of branches on the tree (the ‘test’ set) and all DD clades as the ‘reference’ set. RELAX analysis was executed from the command line using the following command:

hyphy relax --alignment /path/to/data.txt --code Invertebrate-mtDNA --kill-zero-lengths No --test hd --models Minimal.

Our input data were a .fna file containing the aligned nucleotide sequences and the phylogeny with HD lineages labelled as the ‘test’ group. Inferences from RELAX are based on comparisons of *ω*, the ratio of non-synonymous to synonymous substitutions (*K*_a_/*K*_s_). The null hypothesis tested by RELAX is *K* = 1, where *ω*_HD_ = *ω*_DD_*^K^*. RELAX implements a likelihood ratio test that considers the phylogeny to determine if *K* ≠ 1. While RELAX is a well supported model for assessing variation in evolutionary rate, its ability to distinguish relaxed from intensified selection is debated [61,62]. For this reason, we only use RELAX to determine if HD affects the evolutionary rate of COI; we do not use it to draw conclusions about the mechanism underlying rate differences.

### (f) Incidence of indels

The occurrence and placement of indels in the consensus amino acid sequence for each family were determined through comparison with the overall insect consensus sequence. The secondary structure of each amino acid sequence was predicted using SWISS-MODEL [63] and visualized in Jalview [64] with helix and loop numbering following Pentinsaari *et al*. [65]. Figures were refined using Inkscape 1.3.2 (gitlab.com/inkscape/inkscape).

## 3. Results

### (a) Amino acid substitutions in insect cytochrome *c* oxidase subunit I

**Figure 2.**
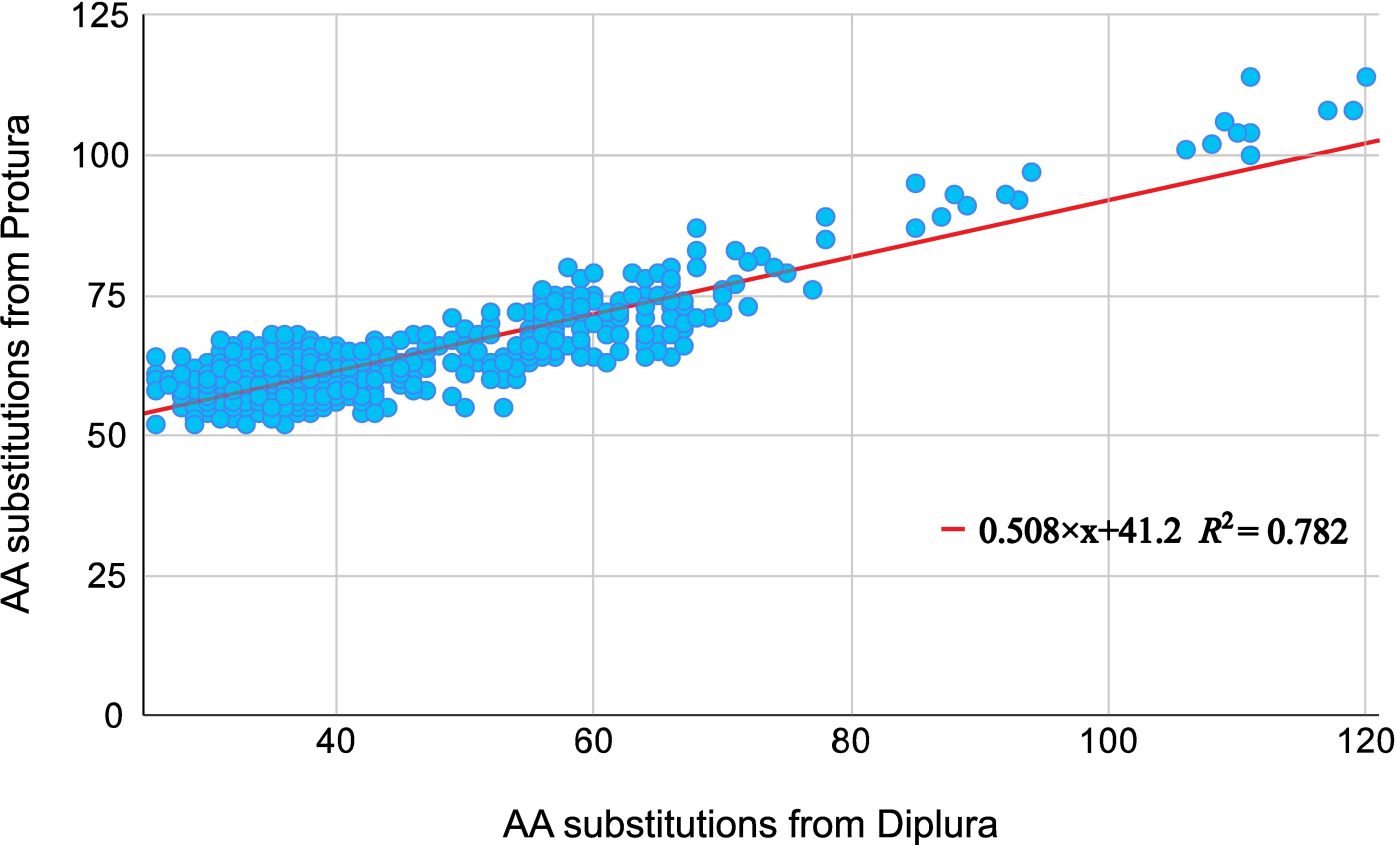
Relationship between the number of amino acid (AA) substitutions for a consensus dipluran sequence and a consensus proturan sequence and the consensus sequence for each of 783 insect families.

The barcode region of COI typically spans 220 codons, and 179 of these positions showed amino acid substitutions among the BINs examined in this study. The number of these substitutions between the dipluran consensus and the consensus sequence for each of the 783 insect families had a greater range (26**–**120) than with the proturan (52**–**114; figure 2). However, the number of substitutions between each insect family and the dipluran/proturan outgroups was strongly correlated (*r*^2^ = 0.78). Because of this congruence (figure 2 and electronic supplementary material, table S3), subsequent analyses only used values from the dipluran comparison (electronic supplementary material, table S4). The two subclasses and three superorders composing the Insecta showed nearly twofold variation in their mean number of amino acid substitutions from the dipluran outgroup (electronic supplementary material, table S5 and figures S2–S11).

### (b) Overview of patterns of amino acid substitution

**Figure 3.**
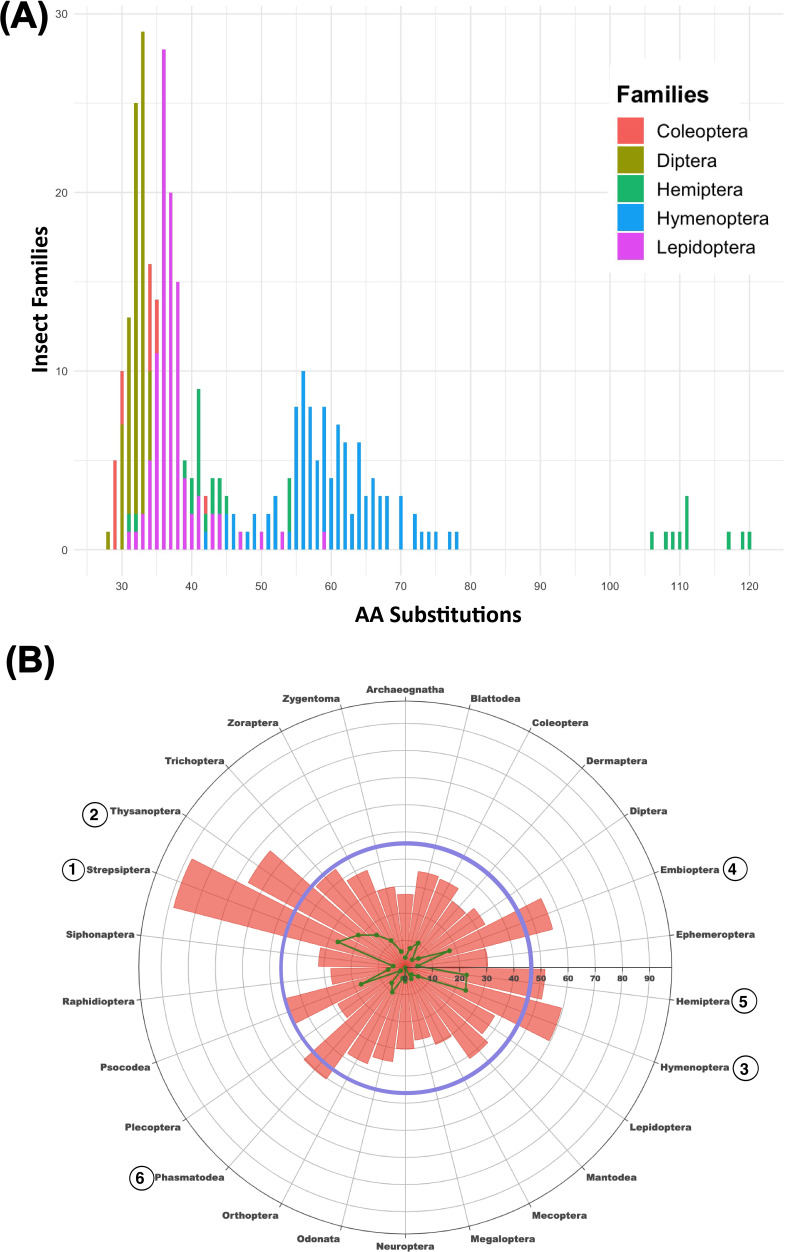
(A) Histogram of the number of amino acid (AA) substitutions between the consensus sequence for a dipluran outgroup and 523 families in the five major orders of insects. (B) Radar plot of the mean number of AA substitutions between a dipluran outgroup and 26 orders of insects. The blue annulus indicates the upper quartile (>46) of substitutions. Interfamily Poisson genetic distance is shown in the green scatter plot.

Across all families (electronic supplementary material, figure S1), the mean number of substitutions from the outgroup was 42. Considering the upper quartile, 20% of families had 46–66 substitutions, 3% had 67–85 substitutions and 2% had more than 86 substitutions. To aid subsequent discussion, we consider families in the upper quartile (>46 substitutions) as having a high number of substitutions, while those in the lowest quartile (<34 substitutions) are classed as having few substitutions. Notable differences within and among insect lineages are considered in electronic supplementary material, results. The mean number of substitutions varied over threefold among families in the five major insect orders (figure 3 and electronic supplementary material, figures S2–S6 and tables S4 and S6).

### (c) Variation in substitutions within insect lineages

Differences in substitution counts were also apparent among and within lineages in each superorder and often associated with life history traits (electronic supplementary material, tables S7–S9). For example, among Psocodea, the 24 free-living, winged families of Psocomorpha, Trogiomorpha and Troctomorpha had few substitutions (*$\bar{x}$* = 37.5), while the nine flightless families (also parasitic) of Phthiraptera had far more (*$\bar{x}$* = 67.7). Most families of Hymenoptera (99/103) were in the upper quartile ($\bar{x}$ = 59.5), although many are both free-living and capable of flight. The Cecidomyiidae was the only dipteran family from 114 to reach the upper quartile, and it is HD. Only two insect orders—Thysanoptera and Hymenoptera—are exclusively HD, and nearly all their component families fell in the upper quartile, although substitution counts did vary among their lineages (electronic supplementary material, figures S1, S5 and S9 and tables S10 and S11). Among the four orders with mixed sex-determination systems, HD lineages also regularly showed more amino acid substitutions than DD lineages.

The 652 DD families exhibited fewer amino acid substitutions ($\bar{x}$ = 37.8) than the 131 HD families ($\bar{x}$ = 63.9) (electronic supplementary material, table S12). The Strepsiptera was a notable exception as its eight families all showed many amino acid substitutions ($\bar{x}$ = 88.2), with a range from 78 (Xenidae) to 94 (Corioxenidae) (electronic supplementary material, figures S1 and S11G and table S4).

### (d) Evidence for rate acceleration in haplodiploid lineages

**Figure 4.**
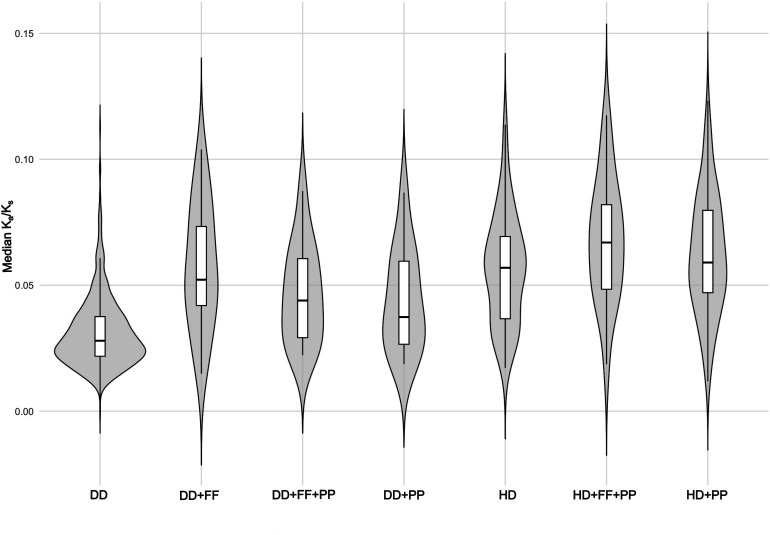
Violin plots of median *K*_a_/*K*_s_ values for 783 insect families grouped by combinations of reproductive mode (DD or HD), locomotion (flight or flightless females—FF) and lifestyle (free-living or parasite/parasitoid—PP). Each violin plot represents the density distribution of values within a group, with an embedded boxplot showing the median and interquartile range.

*K*_a_/*K*_s_ values for the 783 insect families were low, ranging from 0.00 to 0.12, an expected result given the intense purifying selection on COI (electronic supplementary material, table S13). *K*_a_/*K*_s_ values were not related to the species richness of a family (electronic supplementary material, figure S12), but the median *K*_a_/*K*_s_ was twice as high for HD as for DD families (figure 4). A multivariate ANOVA (MANOVA) analysis (table 1 and electronic supplementary material, table S14) further revealed that *K*_a_/*K*_s_ was significantly higher in HD taxa, in parasitic/parasitoid taxa and in flightless lineages, with the highest values in taxa that combined these traits (figure 4).

**Table 1.** Multivariate ANOVA examining the impact of three factors (reproduction—HD or DD; lifestyle—free-living (FL) or parasite/parasitoid (PP); locomotion—winged female (WW) or flightless females (FF)) on *K*_a_/*K*_s_ in 783 insect families. n.s., not significant.

	sum sq.	mean sq.	*F*-value	Pr(>*F*)	significance
reproduction	0.0928	0.0928	346.40	<2 × 10^−16^	<0.0001
lifestyle	0.0063	0.00627	23.42	1.57 × 10^−6^	<0.0001
locomotion	0.0071	0.00711	26.52	3.30 × 10^−7^	<0.0001
reproduction : lifestyle	0.00002	0.00002	0.07	0.7863	n.s.
reproduction : locomotion	0.0017	0.00174	6.51	0.01090	0.01
lifestyle : locomotion	0.0026	0.00263	9.81	0.00179	0.001

Phylogenetic analysis confirmed that the rate of molecular evolution for COI, as quantified by the number of amino acid substitutions, was significantly higher in HD than in DD lineages. RELAX revealed that *ω* (= *K*_a_/*K*_s_) was 3.5× higher in HD than in DD lineages (0.0875 versus 0.0252). Specifically, *K* = 0.24 with a likelihood ratio test value of 1265.1 (*p* < 0.0001).

### (e) Indels in cytochrome *c* oxidase subunit I

**Figure 5.**
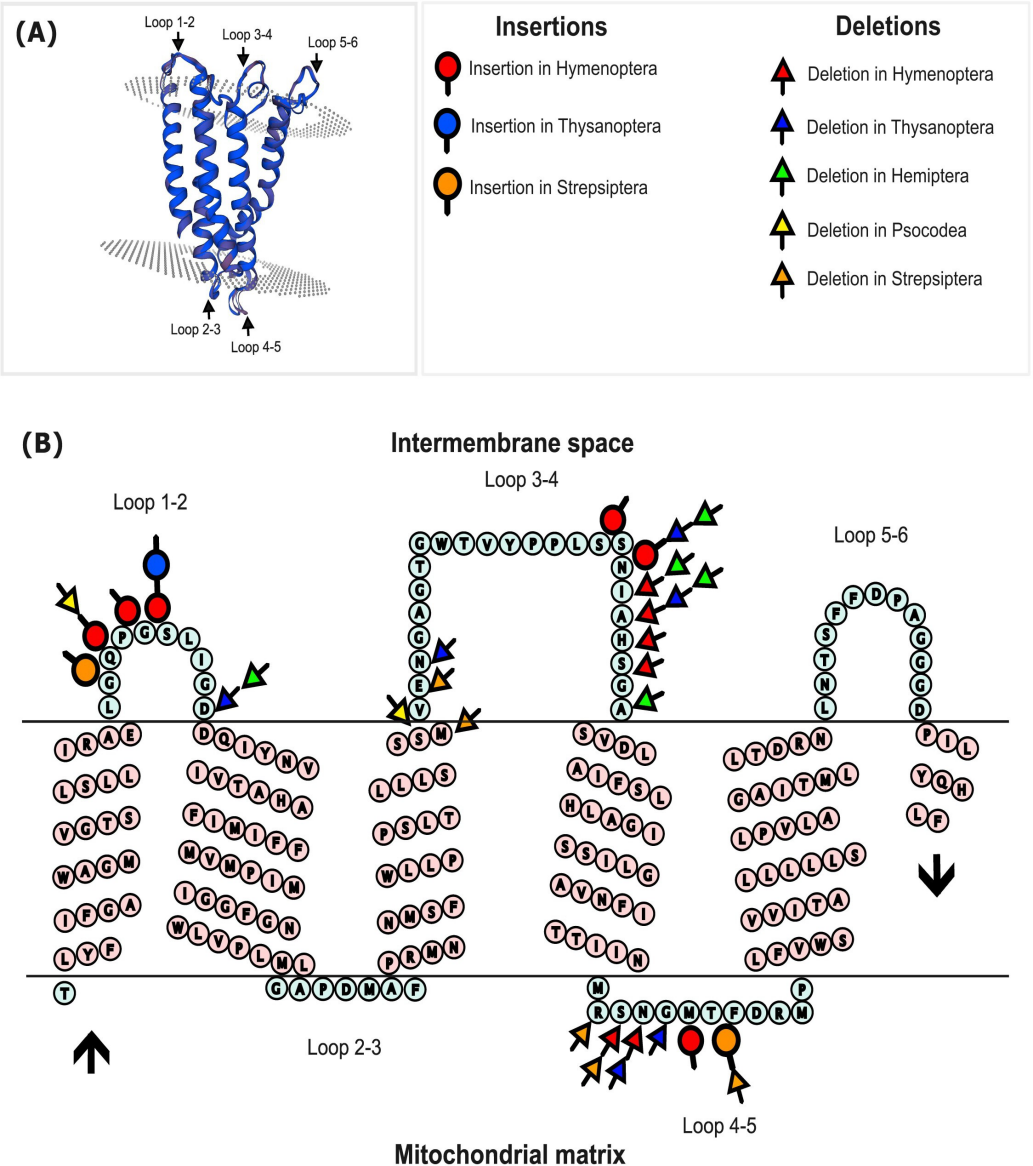
(A) Three-dimensional model of the secondary structure of the protein encoded by the cytochrome *c* oxidase 1 (COI) barcode region. (B) Secondary structure of the consensus amino acid sequence for COI in insects showing the positioning of insertions and deletions in five insect orders. The black arrows mark the N- and the C-termini of COI.

Indels were only detected in 8 of the 652 DD families; all were in Strepsiptera. By contrast, 55 of 131 HD families possessed indels, including taxa in both HD orders (Hymenoptera, Thysanoptera) and in both orders (Hemiptera, Psocodea) with more than one HD family. In total, 69 indels were detected, and these included 11 insertions and 58 deletions (figure 5 and electronic supplementary material, table S15). Consideration of the positioning of these indels, together with their phylogenetic distribution, indicates a minimum of 27 indel events in HD families versus 4 indel events in DD families.

## 4. Discussion

The cytochrome *c* oxidase 1 (COI) protein is critical for oxidative phosphorylation, but its functionality depends upon interactions with the other two COI proteins encoded by the mitogenome plus an array of proteins encoded by the nucleus [66–69]. Although COI has the slowest evolutionary rate of the 12 protein-coding genes in the animal mitogenome [70], the present study revealed amino acid substitutions at 81% of the sites (179/220) in the barcode region. This variation reflects, in part, the fact that our analysis was taxonomically comprehensive; it examined 26 of 29 insect orders, and they included representatives of all five major lineages (Apterygota—2, Palaeoptera—2, Polyneoptera—8, Paraneoptera—3, Holometabola—11). These major lineages themselves showed twofold variation in the number of amino acid substitutions compared with outgroup taxa, and even larger differences were apparent among their component orders and families.

Some taxa showing elevated substitution possess life history traits previously associated with rate acceleration, such as the loss of flight [7] or a parasitic lifestyle (e.g. [6,8,71]). However, additional factors clearly modulated mitogenome evolution. Some flightless taxa (e.g. Apterygota), even those with a parasitic lifestyle (e.g. Siphonaptera), have few substitutions, while other lineages capable of flight and free-living, such as Thysanoptera, have many. This study identifies HD as a key modulator. Nearly all families in the two HD orders (Hymenoptera, Thysanoptera) were in the upper quartile. In the four orders with mixed sex-determination systems, HD taxa showed more amino acid substitutions than DD.

Trait covariation makes it difficult to disentangle the relative impacts of flight loss, parasitism and sex-determination system in accelerating mitogenome evolution. For example, the hemipteran families with the most amino acid substitutions are HD, but their females are also flightless and plant parasites. Likewise, the rapid evolutionary rate in the phthirapteran lineage within Psocodea can be ascribed to the synergistic effect of all three factors. Despite such covariation, MANOVA emphasized the importance of HD in accelerating mitogenome evolution, placing it as a key determinant of *K*_a_/*K*_s_ while controlling for the other traits, a result reinforced by specific cases. For example, 21 families of Diptera are parasitic [49], but only one, the Cecidomyiidae, is in the upper quartile, and it is the only dipteran family known to be exclusively HD. Similarly, among the 16 curculionid subfamilies analysed, the Scolytinae had the highest mean substitution rate, and its HD tribe, Xyloborini, had the most substitutions.

The qualitative evidence for accelerated mitogenome evolution in HD lineages was quantified by statistical analysis. RELAX [23] revealed that HD lineages had 1.7× as many amino acid substitutions as their DD counterparts and 3.5× higher *K*_a_/*K*_s_. When analysis focused on confamilial species, the same pattern was evident as the median *K*_a_/*K*_s_ was 2× higher in HD than DD lineages. These differences are far greater than those in other systems that have attracted attention. For example, high- and low-elevation species show just a 10% difference in *K*_a_/*K*_s_ [24]. While RELAX suggests that selection is relaxed on HD lineages, we emphasize that distinguishing relaxed from intensified selection is challenging.

While amino acid substitutions in key proteins such as COI are likely to influence fitness, the insertion or deletion of amino acids must, on average, have an even greater impact [72–74]. In congruence with genome-wide studies [75], indels in COI were 1–2 codons in length, with deletions about twice as common as insertions. Importantly, indels were restricted to HD taxa except for the Strepsiptera. Members of this order are thought to be DD, but they couple patterns of sequence variation (frequent amino acid substitutions, high *K*_a_/*K*_s_, indels) otherwise seen only in HD taxa. While this congruence suggests that factors favouring amino acid substitutions also facilitate indels, it also raises the need to verify that paternal genome elimination does not occur in Strepsiptera.

While further studies are required to clarify the cause of the accelerated mitogenome evolution in HD taxa, it is unlikely to reflect higher exposure to genetic drift because *N*_e_ of the mitogenome is not directly impacted by the transition to HD. However, males of HD taxa do expose all variation in their nuclear genomes to selection [11]. They may not transmit mitochondria, but they do transmit nuclear genes that foster mitochondrial–nuclear coevolution. By exposing genes with favourable effects on mitochondrial-nuclear coevolution to selection, we hypothesize that haplodiploidy facilitates the fixation of newly arisen mitochondrial mutations that would otherwise be lost to drift or rejected by selection. An additional and not mutually exclusive possibility is that the reduced effective population size (*N*_e_) of nuclear genes—characteristic of HD lineages—increases the likelihood that mildly deleterious mutations in the nuclear genome become fixed. These changes could subsequently impose selective pressure on the mitogenome, promoting the rise of compensatory mutations to preserve critical mito-nuclear interactions. These possibilities highlight the need for a detailed investigation of both processes to disentangle their respective roles in driving the rapid mitogenome evolution characteristic of HD taxa. Muller’s ratchet [76] has also been suggested as the mechanism driving mitochondrial gene rearrangements [77] and the fragmentation of mitogenomes [71,78] in HD lineages. As this capacity would seem particularly valuable in arms races, it is no surprise that HD insects are prominent parasites/parasitoids of both plants and other insects.

Although this study only examined patterns of sequence diversity in 5% of the mitogenome, prior studies have established that the COI barcode region is a strong proxy for the mitogenome [32–34]. As such, elevated rates of non-synonymous substitutions, elevated *K*_a_/*K*_s_, and indels are likely to characterize the mitogenomes of HD species, a prediction supported by genome-wide studies on a few arthropods [12].

Variation in rates of mitogenome evolution may have implications for speciation. Rapid evolution of mitochondrial DNA (mtDNA) has been linked to Dobzhansky–Muller incompatibilities [79], and mitogenome divergence seems a better predictor of reproductive isolation in hybrid zones than nuclear DNA [41]. Qualitative patterns are also consistent with HD impacting speciation. For instance, among all 150+ families of Diptera, the sole HD family (Cecidomyiidae) is, by far, the most speciose [32]. Given the emerging mechanistic links between HD and reproductive isolating barriers, we speculate that sex-determining systems play an important role in modulating speciation.

Future studies that examine whole genomes will undoubtedly advance our understanding of the evolutionary dynamics of mito-nuclear interactions and the impacts of sex-determination systems on their kinetics. The present study incentivizes work to test the generality of rate acceleration by extending analysis to more HD lineages, within both the Arthropoda (e.g. mites) and other animal phyla (e.g. Nematoda, Rotifera). If HD is confirmed to be a general evolutionary accelerant in this kingdom, studies should expand to consider the impacts of haploid/diploid life cycles in fungi, plants and protists. Do lineages whose lifecycle is largely haploid (e.g. mosses) show more rapid plastid evolution than those that are diploid (e.g. higher plants, ferns)? Do plastid genomes evolve more quickly in protistans with haplontic than diplontic life cycles [80]? The examination of these systems will help to clarify the effect of genome structure on evolutionary rates.

## Data Availability

A dataset is availble on Dryad [81]. Supplementary material is available online [82].
